# Polymorphisms in the Interleukin 18 Receptor 1 Gene and Tuberculosis Susceptibility among Chinese

**DOI:** 10.1371/journal.pone.0110734

**Published:** 2014-10-31

**Authors:** Junxian Zhang, Li Zheng, Donglin Zhu, Huiru An, Yourong Yang, Yan Liang, Weiguo Zhao, Wenjun Ding, Xueqiong Wu

**Affiliations:** 1 Army Tuberculosis Prevention and Control Key Laboratory, Institute for Tuberculosis Research, the 309th Hospital of Chinese PLA, Beijing, P. R. China; 2 Laboratory of Environment and Health, College of Life Sciences, University of Chinese Academy of Sciences, Beijing, P. R. China; Duke University, United States of America

## Abstract

Tuberculosis (TB), an infectious disease caused by infection of *Mycobacterium tuberculosis*, is a major public health challenge globally. Genetic epidemiological evidence suggests a genetic basis for TB, but the molecular mechanism for a genetic predisposition to TB remains largely unknown. Thirty-five tag single-nucleotide polymorphisms (SNPs) across 11 candidate cytokines and related genes, including IL-12/IFN-γ axis genes (*IL12B*, *IL12RB1*, *IL18R1*, *IL27, IFNGR1*, *IFNGR2* and *STAT1*), the *TNF* gene locus (*TNF* and *LTA*), *IL10*, and *CCL2*, were genotyped using Sequenom's iPLEX assays in 1,032 patients with TB and 1,008 controls of Chinese Han origin. We did not find that any of the 35 tag SNPs individually or as haplotypes was significantly associated with susceptibility to TB, on the basis of multivariable logistic regression analysis with adjustment for age and sex. However, stratification analyses showed that, in those with age 46 years or older, carrying the rs1974675 T allele in the *IL18R1* gene had a significantly decreased susceptibility to TB occurrence compared with carrying the C/C genotype (OR = 0.57, *P* = 5.0×10^−4^). Further analysis indicated that a SNP in absolute linkage disequilibrium with rs1974675, rs3755276, is located within a CpG dinucleotide and showed hypomethylation in controls than in patients (19.6% vs. 31.4%; *P* = 1.0×10^−4^) and genotype-specific DNA methylation at the *IL18R1* promoter and *IL18R1* mRNA levels. In addition, DNA methylation levels were significantly inversely correlated with mRNA levels. Thus, decreased mRNA levels of *IL18R1* due to rs3755276 may partially mediate the increased susceptibility to TB risk.

## Introduction

Tuberculosis (TB), an infectious disease caused by *Mycobacterium tuberculosis* (*M. tuberculosis*), is a global disease with high burden particularly in Africa and Asia. As of 2010, China has the second-largest number of incident cases worldwide [1]. Despite the availability of effective treatment over the past few decades, TB remains the first leading cause of deaths from infectious diseases worldwide. Multiple factors contribute to TB, including host–pathogen interactions and environmental factors. Furthermore, there has been evidence demonstrating that the risk of developing TB in humans was strongly influenced by genetic factors [2]. Unraveling the mechanisms underlying genetic susceptibility to TB may lead to a better understanding of the pathogenesis of TB and development of novel strategies for prevention and treatment of this disease.

Immune responses to *M. tuberculosis*, including innate and adaptive immune responses, are regulated by interactions between immune cells and the cytokines secreted by these cells [3]. After phagocytosis, *M. tuberculosis* is processed within macrophages and dendritic cells (DCs), and induces the proinflammatory cytokines interleukin (IL)-12, IL-1, and tumor necrosis factor (TNF) [4], [5]. The IL-12/interferon (IFN)-γ axis plays a critical role in initiating a T helper 1 (Th1) T-cell response and protecting against mycobacterial diseases [6]. IL-12 can cause Th1 cells to proliferate and produce IFN-γ. IFN-γ, produced by CD4^+^ T cells (mostly Th1), CD8^+^ T cells and natural killer cells, activates the macrophages, causing them to become microbicidal. TNF induces cytokine and chemokine production by macrophages, activates macrophages for killing, and modulates macrophage apoptosis [7], [8]. During *M. tuberculosis* infection, TNF is important for the cell recruitment required to form granulomas that restrict bacilli replication and prevent bacterial dissemination [9]–[13]. The anti-inflammatory cytokines, such as IL-10, can also be produced by *M. tuberculosis*-infected macrophages, which downregulate proinflammatory cytokines and T-cell proliferation and activation, balancing the response between bacterial eradication and host survival [4], [14]. In addition, chemokines are essential components of the innate immune response against pathogens of the respiratory tract. The C–C chemokine ligand-2 gene (CCL2) encodes the monocyte chemoattractant protein-1 (MCP-1), which recruits immune cells to the site of mycobacterial disease [15]. This protein is also involved in granuloma formation and, through this process, may help to contain *M. tuberculosis* in the lungs [16]. Several single-nucleotide polymorphism (SNP)-based studies have reported associations between many genes, including those involved in the IL-12/IFN-γ axis, TNF, IL-10, and CCL2, and susceptibility to TB in different populations. However, many of these studies have yielded inconsistent results [17], [18].

Here, we conducted a genetic association study in a large case-control population of Chinese Han origin, focusing on IL-12/IFN-γ axis genes, including interleukin 12B (*IL12B*), interleukin 12 receptor beta 1 (*IL12RB1*), interleukin 18 receptor 1 (*IL18R1*), *IL27*, interferon gamma receptor 1 (*IFNGR1*), interferon gamma receptor 2 (*IFNGR2*) and signal transducer and activator of transcription 1, 91kDa (*STAT1*), *TNF* gene locus including *TNF* and lymphotoxin alpha (*LTA*, previously named *TNFB*), *IL10*, and *CCL2*. By testing 35 tag SNPs across these genes, we found that none of the SNPs individually or in haplotype were significantly associated with susceptibility to TB, on the basis of multivariable logistic regression analysis with adjustment for age and sex. However, by conducting age-stratified analyses, we found that in the older group (≥46 years), rs1974675 in the *IL18R1* gene locus showed significant association with susceptibility to TB occurrence. Furthermore, we found that one of the SNPs, rs3755276, is in absolute linkage disequilibrium (LD) with rs1974675 (*r* ^2^ = 1). rs3755276 is located within a CpG dinucleotide and showed genotype-specific methylation and mRNA levels of *IL18R1*. In addition, DNA methylation levels were significantly inversely correlated with mRNA levels. Based on these results, we propose that the genetic association between polymorphisms in the *IL18R1* gene locus and susceptibility to TB among Chinese is probably partially attributed to genotype-specific methylation of polymorphisms in the *IL18R1* promoter and, therefore, genotype-specific modulation of *IL18R1* expression in TB.

## Materials and Methods

### Ethics statement

Written informed consent was obtained from all participants involved in this study, and the study was approved by the Research Ethics Committee of the 309th Hospital of Chinese PLA (Beijing, China).

### Subjects and Samples

A total of 1,032 patients with TB were recruited at the 309th Hospital of Chinese PLA (Beijing, China), between June 2009 and March 2013. All patients with TB were diagnosed according to the criteria: 1) smear or culture positive for *M. tuberculosis* and/or 2) clinical–radiological and histological diagnosed. The male/female ratio was 1.13, and the mean age was 39.3 years (±SD, 19.3) (Table S1). Totally 1,008 controls were randomly selected from patients admitted to the same hospital during the same time period as the TB patients were collected. Controls had no history of TB, with retrospectively confirmed non-tuberculous diseases. Controls were admitted for a wide range of conditions: cardiovascular diseases (26.4%); bone diseases (19.5%); neurological or psychiatric conditions (17.5%); acute upper respiratory infections (14.3%); kidney diseases (8.1%); gastrointestinal or hepatobiliary system complaints (6.2%); and other diseases of blood, urine, lymph, eye, or skin (8.0%). For the 1,008 controls, the male/female ratio was 1.42, and the mean age was 45.2 years (±SD, 24.2) (Table S1).

To examine LD between rs3755276 and rs1974675, and correlate genotypes with methylation and mRNA expression levels, we recruited additional 95 healthy controls during physical examination at the 309th Hospital of Chinese PLA (Beijing, China), between October 2013 and June 2014, with the male/female ratio 1.16, and the mean age 52.1 years (±SD, 5.6) (Table S1).

All participants were unrelated Han Chinese. None of them had a clinical history of diabetes mellitus, HIV infection, or receipt of immunosuppressive therapy.

### Extraction of Genomic DNA

A 2-mL volume of venous blood samples from each participant was taken in citrate-anticoagulated glass tubes, and were frozen at −40°C. Total genomic DNA of the leucocyte was extracted from 1 ml of peripheral blood using the Whole Blood DNA Extraction Kit (Tiangen Biotech, Co., Ltd, Beijing, China), according to the manufacturer's instructions. Genomic DNA extracted was dissolved in 0.1×TE buffer (10 mM Tris - 1 mM EDTA, pH8.0) and stored at −20°C.

### SNP Selection

Tag SNPs were selected using the Haploview 4.2 program (http://www.broad.mit.edu/haploview/; HapMap Phase II+III, release 27, CHB data), with *r^2^*>0.8 with those untyped SNPs and minor allele frequency (MAF) >5%. Totally 35 tag SNPs were selected for the 11 candidate genes.

### SNP Genotyping and DNA Methylation Analysis

The 35 SNPs were genotyped in the 1,032 patients with TB and 1,008 controls using 2 custom-designed assays by MassARRAY (Sequenom, iPLEX platform). Two no-template controls and four duplicated samples were arrayed in each 384-well format as quality controls. All genotyping results were generated and checked by laboratory staff unaware of patient status. Twenty two subjects were excluded for low call rates (<95%). The overall genotyping success rate was 99.1%. For genotyping rs1974675 and rs3755276 in the additional sample set of 95 healthy controls, the Sanger sequencing method was used. Sequences of primers are available on request.

For methylation analysis in the 1,032 patients with TB and the 1,008 controls, we randomly selected six cases and six age- and sex-matched controls in each genotype group (C/C, C/T, or T/T at rs3755276; Table S1). Bisulfite conversion of 1 µg genomic DNA using EZ DNA methylation-Gold Kit (Zymo) was performed according to manufacturer's instructions. Primers were designed using the EpiDesigner tool (Sequenom) and sequences of primers are available on request. Hot-start PCR was used to amplify the *IL18R1* promoter from the bisulfite-converted genomic DNA. Then MassARRAY MALDI-TOF mass spectrometry–based quantitative DNA methylation analysis was carried out in triplicate by standard protocol (Sequenom EpiTYPER platform).

For methylation analysis in the additional sample set of 95 healthy controls, we randomly selected 17 individuals from those carrying C/C, randomly selected 10 individuals from those carrying C/T, and selected all of the 3 individuals carrying T/T genotypes at rs3755276 (Table S1). Bisulfite conversion of 1 µg genomic DNA using EZ DNA methylation-Gold Kit (Zymo) was performed. Primers were designed using the Pyrosequencing Assay Design software (Biotage, Uppsala, SW) and sequences of primers are available on request. Hot-start PCR was used to amplify the *IL18R1* promoter from the bisulfite-converted genomic DNA. Then the amplicons were subjected to quantitative pyrosequencing in triplicate in a Biotage PSQ 96MA system by protocols suggested by the supplier of the instrument.

### Real-time RT-PCR

RNA templates were extracted from blood lymphocyte cells of the 30 healthy controls used in methylation analysis with the RNeasy miniprep kit (Qiagen). cDNAs were made from mRNA templates using a standard reverse transcriptase protocol (iScript, BioRad) by use of 500 ng of RNA per reaction. Quantitative RT-PCR for IL18R1 was carried out in triplicate by the SYBR Green method on an iQ5 real-time PCR detection system (BioRad) using iQ SYBR Green Supermix (BioRad catalog n° 170–8862). Normalization for mRNA quantity was performed with human GAPDH control primers for each sample and final abundance figures adjusted to yield an arbitrary value of 1 for rs3755276 C/C homozygotes using the ΔΔCt method. Melting curve analyses were performed to rule out non-specific amplification. Primers used for the amplification of IL18R1 and GAPDH are available on request.

### Statistical Analyses

Comparisons of sex and age between TB patients and controls were performed using the *χ^2^* test. Differences of mean age between cases and controls were analyzed by use of an unpaired *t* test. Logistic regression under dominant, recessive, and additive inheritance models were used to calculate *P* values, Odds ratios (ORs), and 95% confidence intervals (CIs) for assessing the association between SNPs and disease risk, adjusting for age and sex. Due to the limited sample size, age- or sex-stratified analyses of SNPs were based on a dominant inheritance model. In age-stratified analyses of SNPs, we further adjusted for age (continuous), together with sex, in the younger group and the older group respectively, to eliminate the residual confounding derived from age-unmatching between cases and controls in each group. In the additional healthy controls, two-way ANOVA was used to compare the mean age among the three genotypic groups. Comparisons of sex distribution among the three genotypic groups were performed by use of the *χ^2^* test for 2×3 contingency tables. Differences of methylation levels and mRNA levels between groups were analyzed using unpaired *t* test. A Pearson's test was used to calculate the correlation coefficient (*ρ*) between mRNA levels and DNA methylation. All tests are two-tailed. These analyses together with Hardy-Weinberg equilibrium (HWE) and haplotype analyses were performed using the online software SNPStats (http://bioinfo.iconcologia.net/snpstats/) and SPSS (version 10.0; SPSS).The population-attributable fraction (PAF) associated with genetic risk factors was estimated by the formula PAF = f (RR-1)/[1+f (RR-1)], where f is the population exposure rate, and RR is the relative risk [20].

## Results

Thirty-five selected tag SNPs in *IL12B, IL12RB1, IL18R1, IL27, IFNGR1, IFNGR2, STAT1, TNF, LTA, IL10,* and *CCL2* genes were genotyped in 1,032 TB cases and 1,008 controls of Chinese Han origin, with all genotype distributions in the control group consistent with those expected from the HWE (*P* values >0.01). In the single-locus analyses, none of the 35 SNPs in the 11 candidate genes were significantly associated with susceptibility to TB in the Chinese population, with *P* values greater than 0.01 on the basis of multivariable logistic regression analysis with adjustment for age and sex (Table S2). In addition, haplotype analysis was performed for every candidate gene. We identified 2 to 5 common haplotypes with frequencies greater than 10% in each of the 11 candidate genes, but none of them showed a significant association with TB risk (*P* values >0.01; data not shown).

**Figure 1:**
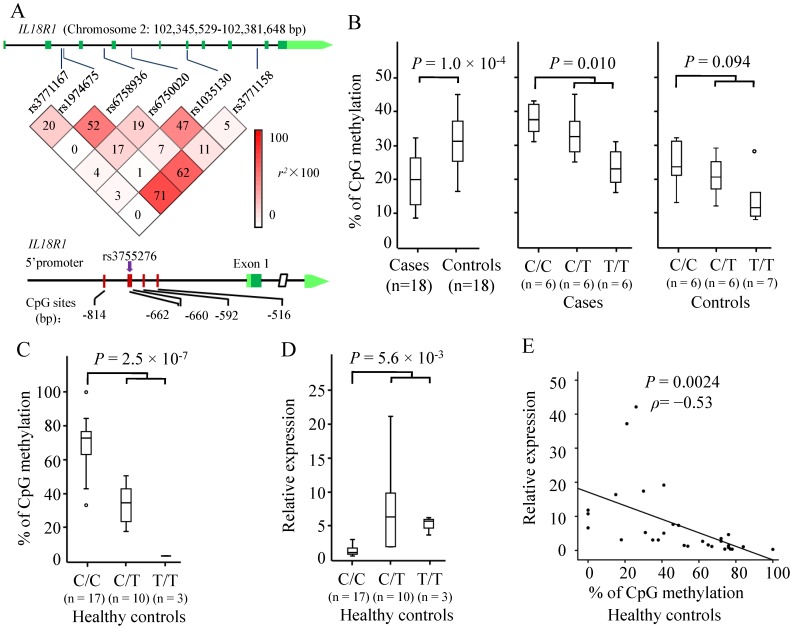
SNPs in the *IL18R1* gene, DNA methylation at the *IL18R1* promoter and *IL18R1* mRNA levels. (A) The upper panel indicates the tag SNPs across the *IL18R1* gene and the linkage disequilibrium (LD) structure in the Chinese case-control population. Pairwise LD (measured by *r* ^2^) between SNPs is indicated by the color of individual squares in the triangular graphic, the squares with the deepest color have *r* ^2^ = 1. The lower panel indicates the five CpG sites at the *IL18R1* promoter (−814, −662, −660, −592, and −516 bp), where a SNP in absolute LD with rs1974675 (*r^2^* = 1 in CHB), rs3755276, is located within the second CpG site (−662 bp). Position +1 is determined by the start codon. (B) Correlation between methylation status at two CpG sites next to each other (−662 and −660 bp) and genotypes at rs3755276 in 18 patients with TB and 18 controls. DNA methylation was analysed using the EpiTYPER platform (Sequenom) which recognizes the two CpG sites (−662 and −660 bp) as one methylation signal. (C) Correlation between methylation status of the CpG site at −662 bp and genotypes at rs3755276 in additional 30 healthy controls. DNA methylation was analysed at a single nucleotide level by quantitative pyrosequencing in a Biotage PSQ 96MA system. (D) Correlation between *IL18R1* mRNA levels and genotypes at rs3755276 in the 30 healthy controls. (E) Correlation between *IL18R1* mRNA levels and DNA methylation at −662 bp (rs3755276) in the 30 healthy controls. A Pearson's test was used, and the correlation coefficient (*ρ*) and the two-tailed significance are shown. Outliers are marked with a circle on the boxplot. Error bars indicate means ±SD.

The stratification had no significant effects on the results obtained in analyses stratified for sex (*P* values for heterogeneity >0.01; data not shown). However, when conducting age-stratified analyses on the basis of mean age in controls (<46 or ≥46 years), we found evidence of heterogeneity for two SNPs in the *IL18R1* gene locus (rs1974675 and rs6758936, Table 1, Fig. 1A and Table S3). In the older group, the individuals carrying the rs1974675 T allele had a significantly decreased susceptibility to TB compared with those carrying the C/C genotype (OR = 0.57; 95% CI = 0.41–0.79, *P* = 0.00050); for rs6758936, the individuals carrying the A allele had a significantly decreased susceptibility to TB compared with those carrying the G/G genotype (OR = 0.66; 95% CI = 0.47–0.88, *P* = 0.0053). The association of rs1974675 remained significant even after Bonferroni correction for multiple testing. By contrast, the associations were not significant in younger groups. Case-only analysis in the older patients with TB showed no significant difference on age at TB diagnosis for the protective allele carriers and the at-risk homozygotes (*P* = 0.46 and *P* = 0.73 for rs1974675 and rs6758936, respectively; Table S4).

**Table 1:** Age-stratified association of rs1974675 and rs6758936 in the *IL18R1* gene with TB risk among Chinese TB patients and controls.

Age	Genotype	Cases, n (%)	Controls, n (%)	OR (95% CI) a	*P* a
rs1974675					
≥46 year					
	C/C	302 (81.8)	399 (71.5)	1	
	C/T	62 (16.8)	148 (26.5)	0.56 (0.40–0.79)	0.00076
	T/T	5 (1.4)	11 (2.0)	0.59 (0.20–1.76)	0.35
	C/T-T/T	67 (18.2)	159 (28.5)	0.57 (0.41–0.79)	0.00050
<46 year					
	C/C	479 (73.9)	329 (74.8)	1	
	C/T	152 (23.5)	104 (23.6)	1.01 (0.75–1.35)	0.96
	T/T	17 (2.6)	7 (1.6)	1.71 (0.68–4.26)	0.25
	C/T-T/T	169 (26.1)	111 (25.2)	1.05 (0.79–1.39)	0.74
^ b^ *P*heterogeneity = 0.0034					
rs6758936					
≥46 year					
	G/G	284 (77.6)	382 (69.3)	1	
	G/A	78 (21.3)	157 (28.5)	0.66 (0.48–0.91)	0.011
	A/A	4 (1.1)	12 (2.2)	0.43 (0.14–1.37)	0.15
	G/A-A/A	82 (22.4)	169 (30.7)	0.65 (0.47–0.88)	.0053
<46 year					
	G/G	461 (71.6)	330 (75.7)	1	
	G/A	168 (26.1)	99 (22.7)	1.20 (0.90–1.61)	0.12
	A/A	15 (2.3)	7 (1.6)	1.72 (0.68–4.36)	0.25
	G/A-A/A	183 (28.4)	106 (24.3)	1.24 (0.93–1.64)	0.14
^ b^ *P*heterogeneity = 0.0020					

Due to genotyping failure, the actual sample size for rs1974675 is 1,017 and 998 for the cases and controls, respectively; the actual sample size for rs6758936 is 1,010 and 987 for the cases and controls, respectively. OR, odds ratio; CI, confidence interval.

a The ORs and *P* values were calculated by logistic regression and adjusted for age and sex where appropriate within the strata.

b For difference in ORs within each stratum.

Independent tests in the older group showed that, in a multiple logistic regression analysis, the signal from rs6758936 did not survive when adjusted for the effect of rs1974675 (residual *P* value  = 0.67). On the contrary, rs1974675 remained significant when adjusted for rs6758936 (residual *P* value  = 0.027). These results suggest that there may be a single susceptibility locus in the *IL18R1* gene, and the significance is more likely due to rs1974675.

To mine the functional SNPs affecting susceptibility to TB risk, we used the SNPinfo programme (http://snpinfo.niehs.nih.gov/) and obtained 20 SNPs in strong linkage disequilibrium (LD) with rs1974675 (*r^2^*>0.8). Although none of these SNPs were predicted to alter the transcriptional factor binding site, splicing, or the miRNA binding site (Table S5), DNA sequence analysis revealed that one SNP among them, rs3755276 (C-662T) in absolute LD with rs1974675 in CHB (*r^2^* = 1), is located within a CpG dinucleotide of the *IL18R1* promoter with the disease-susceptible allele C composing a CpG island, suggesting potential functional consequences by altering DNA methylation at CpG sites (Fig. 1A). Although rs3755276 cannot be directly genotyped in TB cases and controls due to DNA exhaustion, the absolute LD value (*r^2^* = 1 in CHB) between rs3755276 and rs1974675 was confirmed in an independent sample set of 95 healthy controls by Sanger sequencing (Table S1). We determined the status of methylation in five CpG sites in the *IL18R1* promoter (−814, −662, −660, −592, and −516 bp), where two CpG sites next to each other (−662 and −660 bp) were recognized as one methylation signal by the EpiTYPER platform. We found that CpG sites in −662 and −660 bp were significantly hypomethylated in 18 controls compared to 18 TB patients (19.6% vs. 31.4%; *P* = 1.0×10^−4^; Fig. 1B), whereas the remaining three CpG sites showed similar methylation levels between cases and controls (data not shown). In particular, the methylation degree of the two CpG sites (−662 and −660 bp) was greater in individuals carrying C/C genotypes than in those carrying C/T and T/T genotypes (*P* = 0.010 and *P* = 0.094 for TB patients and controls, respectively; Fig. 1B). By an independent platform quantifying DNA methylation at a single nucleotide level (Biotage PSQ 96MA), we confirmed that genotype-specific methylation existed at −662 bp (rs3755276), rather than −660 bp, in independent 30 healthy individuals (C/C vs. C/T and T/T, 68.1% vs. 24.5%; *P* = 2.5×10^−7^; Fig. 1C). The CpG site at −660 bp showed 97∼100% methylation levels in these samples (data not shown).

Previously, expression quantitative trait loci (eQTL) analyses have been carried out for primary DCs from 65 individuals of European descent [19]. The results showed that rs10192157, in absolute LD with rs3755276 and rs1974675 (*r* ^2^ = 1), was the most significant *cis*-eQTL SNP associated with the change in expression of *IL18R1* mRNA after *M. tuberculosis* infection (*P* = 0.0011), with the at-risk C/C genotype being associated with decreased *IL18R1* mRNA levels [19]. By contrast, mRNA expressions *of IL1R1* and *IL18RAP* in the same gene cluster were similar across genotypes at rs10192157 [19]. Given the mRNAs of the 30 healthy individuals were available in this study, we further related genotypes of rs3755276 with expression of *IL18R1* mRNA, and confirmed that the at-risk C/C genotype was associated with decreased *IL18R1* mRNA levels (*P* = 5.6×10^−3^; Fig. 1D). In addition, DNA methylation levels were significantly inversely correlated with mRNA levels (*P* = 0.0024, Pearson correlation coefficient *ρ* = −0.53; Fig. 1E).

## Discussion

To our knowledge, this is the first report associating SNPs of the *IL18R1* gene with TB risk in Chinese older people (≥46 years), confirming the initial hypothesis that the *IL18R1* related pathway may play a role in the pathogenesis of TB. In addition, our findings showed, for the first time, that these SNPs in the *IL18R1* promoter were associated with genotype-specific methylation status and that DNA methylation levels of the *IL18R1* promoter were significantly negatively correlated with *IL18R1* mRNA levels. Together with the observation that rs3755276 in the *IL18R1* promoter were associated with genotype-specific *IL18R1* expression, which was supported by previous eQTL analyses [19] and also observed in this study, our results suggest decreased mRNA levels of *IL18R1* due to rs3755276 may partially mediate increased susceptibility to TB risk.

*IL18R1* encodes the IL-18 receptor α (IL-18Rα), which belongs to the IL-1 receptor family. IL-18Rα is expressed in respiratory epithelium, endothelial cells, natural killer cells, macrophages, neutrophils, DCs, B cells and Th1 but not Th2 cells, and induces downstream signals leading to the activation of NF-κB [21], [22]. Expression of IL-18Rα is induced by an IL-12/Stat4 pathway and inhibited by an IL-4/Stat6 pathway in mice [23], [24]. Coupled with the IL-18Rβ chain, IL-18Rα forms an IL-18 signaling complex that activates IFN-γ expression, which is fundamental in the control of *M. tuberculosis* infection [3]. IL-18 strongly synergizes with IL-12 in the induction of IFN-γ, providing additional Th1 effector functions and contributing to polarized immune responses in *M. tuberculosis* infection [25]. IL-18Rα may have additional IL-18-independent functions in TB that IL-18R signaling on antigen-presenting cells was critical for the secretion of IL-23p40 and the subsequent maintenance of IL-17-secreting T cells [26], and the IL-23/IL-17 axis also contributes to IFN-γ production in the control of *M. tuberculosis* infection [27].

The *IL18R1* gene resides on chromosome 2q12.1 together with IL-1 receptor-like 1 (*IL1RL1*) and IL-18 receptor accessory protein (*IL18RAP*). Previous studies have shown that SNPs in the *IL1RL1*-*IL18R1-IL18RAP* gene cluster were associated with a number of immune inflammatory conditions and lung diseases. However, little is known about their impact on TB risk in the Chinese population, though a recent report indicated no significant association in a small Japanese population [28].

In this study, we assessed the associations of 35 tag-SNPs in 11 cytokine and related genes with the risk of occurrence of TB in a large case-control population of Chinese Han origin. We found that none of the SNPs individually or in haplotype were significantly associated with susceptibility to TB, on the basis of multivariable logistic regression analysis with adjustment for age and sex. However, by conducting age-stratified analyses, we found that T allele carriers (C/T and T/T genotypes) of rs1974675 in the *IL18R1* gene, were associated with decreased susceptibility to TB compared with C/C carriers in those with age 46 years or older. Further analysis indicated that one of the SNPs in absolute LD with rs1974675 (*r* ^2^ = 1 in CHB and the 95 individuals in this study), rs3755276, is located within a CpG dinucleotide in the *IL18R1* promoter.

DNA hypomethylation at CpG sites in the promoter region is a well-defined epigenetic phenomenon generally associated with active gene expression [29], for DNA methylation may have both direct effects, by decreasing the binding of trans-acting factors, and indirect effects, by recruiting methyl binding domain proteins that can recruit additional factors to potentiate a repressive chromatin structure [30], [31]. Furthermore, previous studies have established that DNA methylation is clearly required for appropriate expression of cytokines in Th subsets. Interestingly, studies in a mouse model have shown that Stat4 binds directly to the *Il18r1* locus following IL-12 stimulation, transiently increases acetylation of the locus and decreases Dnmt association and DNA methylation of the *Il18r1* promoter, resulting in higher expression of IL-18Rα in Th1 cells [23], [24]. The present study further observed DNA methylation of the *IL18R1* promoter in peripheral blood leukocytes of cases was significantly higher than that in leukocytes of controls, with the at-risk C/C genotype showing increased DNA methylation compared to C/T and T/T genotypes. The genotype-specific methylation was confirmed in an independent sample set by an independent platform quantifying DNA methylation. In addition, rs3755276 in the *IL18R1* promoter was associated with genotype-specific *IL18R1* expression, which was supported by previous eQTL analyses [19]. Accordingly, we found that DNA methylation levels in the *IL18R1* promoter were significantly negatively correlated with *IL18R1* mRNA levels.

Given the role of *IL18R1* in the development of TB, together with the above described functional relevance of rs3755276 in modulation of *IL18R1* methylation, one might expect that individuals who carry the at-risk C/C genotype, and thus probably have partially increased methylation and decreased expression of *IL18R1* and subsequently decreased anti-*M. tuberculosis* function, may be at a higher susceptibility for developing TB. However, further research is needed to associate rs3755276 directly, rather than the proxy rs1974675, with TB risk, and to elucidate the exact mechanism fully. Furthermore, we were not able to dissect the association to the extent of being able to clearly implicate *IL18R1* over the other two genes at the *IL1RL1*-*IL18R1-IL18RAP* locus. *IL1RL1* encodes the receptor for IL-33, and *IL18RAP* encodes IL-18 receptor accessory protein and is required for the high affinity binding of IL-18 to its receptor complex.

If carrying the rs1974675 C/C genotype is regarded as a risk factor for the development of TB, then the PAF associated with this genetic risk factor - the parameter that combines the strength of the epidemiological influence (relative risk) and the frequency of the genotype (population exposure rate) – can be calculated by the RR (OR, 1.75 [95% CI, 1.27–2.44]) combined with the frequency of C/C genotype (71.5%) (Table 1), indicating that 34.9% (95% CI, 16.2–50.7%) of elevation in the risk of developing TB can be attributed to the susceptible effect of the C/C genotype. The PAF value suggests that other genes are likely to modify the susceptibility to this disease in addition to *IL18R1*. With more susceptibility loci being identified (e.g., by genome-wide association studies), and interaction effects among such loci together with other TB risk factors taken into account, the prediction of TB occurrence may become more accurate and clinically usable.

In the present study, we observed a significant interaction between rs1974675 and age in susceptibility to TB, and rs1974675 showed a significant association signal only in older individuals. Similarly, previous studies have reported an age-dependent association between pulmonary tuberculosis and SNPs in genes including *IFNGR2*, *TOX* and *NRAMP1* [32]–[34]. Increased susceptibility to TB disease in the elderly has been linked to waning immune functions, but could also be associated with co-morbid conditions and impaired mechanical lung function [35]–[37]. Meanwhile, the younger patients developed active TB soon after an initial infection [38]. In industrialized countries, the elderly population continues to consistently have the highest age-specific incidence rates for TB each year, indicating a greater effect of the resistant allele of *IL18R1*, given there are more elderly patients. In addition, the age-dependent predisposition to TB conferred by *IL18R1* genotypes adds to the explanations for why mice deficient in the IL-18 receptor were not more susceptible to *M. tuberculosis* when compared to wild-type mice without considering age effects [39], [40].

There are several potential limitations in this study. First, our controls may include asymptomatic individuals with latent TB infection, because tuberculin testing was not performed for every control. Considering the two-stage process of infection with the pathogen and progression to disease, we cannot directly specify which stage of TB was more affected by *IL18R1*. Second, basic characteristics such as smoking and alcohol drinking status were not available in this study, which might cause selection bias and confounding bias. Third, in addition to SNPs in *IL18R1*, we found nominally significant associations with TB for SNPs in *STAT1*, *LTA*, and *IL12RB1* (Table S2). Although these SNPs did not survive the conservative Bonferroni correction, we cannot exclude the probability of false-negative associations. Therefore, larger multicentre studies are required to further confirm our association findings.

In conclusion, in this large case-control association study, we identified that *IL18R1* polymorphisms were associated with the risk of TB in Chinese older people (≥46 years). In addition, SNPs in the *IL18R1* promoter were associated with genotype-specific methylation status and genotype-specific *IL18R1* expression, which together suggest that increased DNA methylation and decreased mRNA expression of *IL18R1* due to the SNP may partially mediate the increased susceptibility to TB risk. Our findings have contributed to the understanding of the mechanisms underlying the genetic predisposition to TB among Chinese.

## Supporting Information

Table S1 N.S., not significant. Cases are patients with tuberculosis, while 1,008 controls are those with non-tuberculous diseases, including cardiovascular diseases (26.4%), bone diseases (19.5%), neurological or psychiatric conditions (17.5%), acute upper respiratory infections (14.3%), kidney diseases (8.1%), gastrointestinal or hepatobiliary system complaints (6.2%), and other diseases of blood, urine, lymph, eye, or skin (8.0%). For controls used in methylation analysis, six C/C carriers had cardiovascular (n = 2), bone (n = 1), neurological (n = 1), respiratory (n = 1), or skin (n = 1) diseases; six C/T carriers had cardiovascular (n = 1), bone (n = 2), neurological (n = 2), or blood (n = 1) diseases; six T/T carriers had cardiovascular (n = 2), bone (n = 2), gastrointestinal (n = 1), or skin (n = 1) diseases. Comparisons of sex and age distributions between cases and controls were performed by use of the *χ^2^* test. Differences of mean age between cases and controls were analyzed by use of an unpaired *t* test. In the additional healthy controls, two-way ANOVA was used to compare the mean age among the three genotypic groups. Comparisons of sex distribution among the three genotypic groups were performed by use of the *χ^2^* test for 2×3 contingency tables.
(DOCX)

Table S2 Chr, chromosome; OR, odds ratio; CI, confidence interval. ^a^ Genomic position (NCBI Build 36). ^b^ Major allele/minor allele. ^c^ Number of minor homozygotes/number of heterozygotes/number of major homozygotes. *P* values, ORs and 95% CIs were calculated under dominant ^d^, recessive ^e^, and additive ^f^ genetic models by logistic regression while adjusting for age and sex. *P* value, OR and 95% CI were not available in some situations due to low frequency of SNPs.
(DOCX)

Table S3 Chr, chromosome; OR, odds ratio; CI, confidence interval. ^a^ Major allele/minor allele. ^b^ Number of minor homozygotes/number of heterozygotes/number of major homozygotes. ^c^ *P* values, ORs and 95% CIs were calculated under dominant genetic models by logistic regression while adjusting for age and sex. ^d^ *P*_heterogeneity_ were calculated to compare the difference of ORs within each stratum of age (<46 and ≥46 years).
(DOCX)

Table S4 ^a^ Differences of mean age between the protective allele carriers and the at-risk homozygotes were analyzed by use of an unpaired *t* test. The protective alleles are T and A for rs1974675 and rs6758936 respectively.
(DOCX)

Table S5 When loaded with an index SNP, SNPinfo can include SNPs with strong linkage disequilibrium (LD) with it and predicts those that may affect following biological functions with alternative allele: transcriptional regulation by affecting transcription factor binding sites (TFBS) activity; premature termination of amino-acid sequence (stop codons); changing of splicing pattern or efficiency by disrupting splice site, exonic splicing enhancers (ESE) or silencers (ESS); alteration of protein structures or properties by changing single amino acids or changing the frame of the protein-coding region; regulation of protein translation by affecting microRNA (miRNA) binding sites activity, and etc (http://snpinfo.niehs.nih.gov/snpinfo). None of the five coding SNPs in strong LD (*r^2^*>0.8, CHB data) with rs1974675 in this locus were predicted functional. None of the fifteen non-coding SNPs were predicted to alter transcriptional factor binding site, splicing, or miRNA binding site.
(DOCX)
