# Supplementary material for: Polymorphisms in the Interleukin 18 Receptor 1 Gene and Tuberculosis Susceptibility among Chinese
Source: PLoS One. 2014 Oct 31;9(10):e110734. doi: 10.1371/journal.pone.0110734 (PMC4216003; doi:10.1371/journal.pone.0110734)
Supplement: Table S1 — N.S., not significant. Cases are patients with tuberculosis, while 1,008 controls are those with non-tuberculous diseases, including cardiovascular diseases (26.4%), bone diseases (19.5%), neurological or psychiatric conditions (17.5%), acute upper respiratory infections (14.3%), kidney diseases (8.1%), gastrointestinal or hepatobiliary system complaints (6.2%), and other diseases of blood, urine, lymph, eye, or skin (8.0%). For controls used in methylation analysis, six C/C carriers had cardiovascular (n = 2), bone (n = 1), neurological (n = 1), respiratory (n = 1), or skin (n = 1) diseases; six C/T carriers had cardiovascular (n = 1), bone (n = 2), neurological (n = 2), or blood (n = 1) diseases; six T/T carriers had cardiovascular (n = 2), bone (n = 2), gastrointestinal (n = 1), or skin (n = 1) diseases. Comparisons of sex and age distributions between cases and controls were performed by use of the χ2 test. Differences of mean age between cases and controls were analyzed by use of an unpaired t test. In the additional healthy controls, two-way ANOVA was used to compare the mean age among the three genotypic groups. Comparisons of sex distribution among the three genotypic groups were performed by use of the χ2 test for 2×3 contingency tables. (DOCX) [file pone.0110734.s001.docx]

**Table S1.** The clinical characteristics of cases and controls included in the study.

| Characteristics | Cases | Controls | *P* |
| --- | --- | --- | --- |
| TB cases and controls |  |  |  |
| All | n = 1032 | n = 1008 |  |
| Age, mean in years ±SD | 39.3 ± 19.3 | 45.2 ± 24.2 | 1.10 × 10^-9^ |
| < 46, n (%) | 441 (43.8) | 660 (64.0) | 5.5 × 10^-20^ |
| ≥ 46, n (%) | 567 (56.2) | 372 (36.0) |  |
| Gender |  |  |  |
| Female, n (%) | 474 (47.0) | 427 (41.4) | 0.010 |
| Male, n (%) | 534 (53.0) | 605 (58.6) |  |
| Used in methylation analysis | n = 18 | n = 18 |  |
| Age, mean in years ±SD | 39.6 ± 17.4 | 40.8 ± 22.3 | N.S. |
| rs3755276 C/C | 40.8 ± 20.3 | 40.3 ± 24.1 | N.S. |
| rs3755276 C/T | 40.5 ± 18.7 | 40.3 ± 18.2 | N.S. |
| rs3755276 T/T | 37.5 ± 15.9 | 41.8 ± 27.9 | N.S. |
| Gender, number of male / female | 13 / 5 | 13 / 5 | N.S. |
| rs3755276 C/C | 4 / 2 | 4 / 2 | N.S. |
| rs3755276 C/T | 4 / 2 | 5 / 1 | N.S. |
| rs3755276 T/T | 5 / 1 | 4 / 2 | N.S. |
|  |  |  |  |
| Additional healthy controls |  |  |  |
| All |  | n = 95 |  |
| Age, mean in years ±SD |  | 52.1 ± 5.6 |  |
| rs3755276 C/C |  | 51.8 ± 5.2 | N.S. |
| rs3755276 C/T |  | 53.3 ± 6.6 |  |
| rs3755276 T/T |  | 52.3 ± 6.4 |  |
| Gender, number of male / female |  | 51 / 44 |  |
| rs3755276 C/C |  | 36 / 32 | N.S. |
| rs3755276 C/T |  | 13 / 11 |  |
| rs3755276 T/T |  | 2 / 1 |  |
| Used in methylation analysis |  | n = 30 |  |
| Age, mean in years ±SD |  | 53.6 ± 6.0 |  |
| rs3755276 C/C |  | 53.2 ± 5.8 | N.S. |
| rs3755276 C/T |  | 54.7 ± 6.7 |  |
| rs3755276 T/T |  | 52.3 ± 6.4 |  |
| Gender, number of male / female |  | 16 / 14 |  |
| rs3755276 C/C |  | 9 / 8 | N.S. |
| rs3755276 C/T |  | 5 / 5 |  |
| rs3755276 T/T |  | 2 / 1 |  |

N.S., not significant. Cases are patients with tuberculosis, while 1,008 controls are those with non-tuberculous diseases, including cardiovascular diseases (26.4%), bone diseases (19.5%), neurological or psychiatric conditions (17.5%), acute upper respiratory infections (14.3%), kidney diseases (8.1%), gastrointestinal or hepatobiliary system complaints (6.2%), and other diseases of blood, urine, lymph, eye, or skin (8.0%). For controls used in methylation analysis, six C/C carriers had cardiovascular (n = 2), bone (n = 1), neurological (n = 1), respiratory (n= 1), or skin (n = 1) diseases; six C/T carriers had cardiovascular (n = 1), bone (n = 2), neurological (n = 2), or blood (n = 1) diseases; six T/T carriers had cardiovascular (n = 2), bone (n = 2), gastrointestinal (n = 1), or skin (n = 1) diseases. Comparisons of sex and age distributions between cases and controls were performed by use of the *χ^2^* test. Differences of mean age between cases and controls were analyzed by use of an unpaired *t* test. In the additional healthy controls, two-way ANOVA was used to compare the mean age among the three genotypic groups. Comparisons of sex distribution among the three genotypic groups were performed by use of the *χ^2^* test for 2 × 3 contingency tables.
