# Supplementary material for: Polymorphisms in the Interleukin 18 Receptor 1 Gene and Tuberculosis Susceptibility among Chinese
Source: PLoS One. 2014 Oct 31;9(10):e110734. doi: 10.1371/journal.pone.0110734 (PMC4216003; doi:10.1371/journal.pone.0110734)
Supplement: Table S2 — Chr, chromosome; OR, odds ratio; CI, confidence interval. a Genomic position (NCBI Build 36). b Major allele/minor allele. c Number of minor homozygotes/number of heterozygotes/number of major homozygotes. P values, ORs and 95% CIs were calculated under dominant d, recessive e, and additive f genetic models by logistic regression while adjusting for age and sex. P value, OR and 95% CI were not available in some situations due to low frequency of SNPs. (DOCX) [file pone.0110734.s002.docx]

**Table S2.** Association analysis of 35 tag SNPs in 11 candidate genes under dominant, recessive and additive genetic models.

| Gene | SNP_ID | Chr | Position ^a^ | Genic location | Allele ^b^ | Cases ^c^ | Controls ^c^ | OR (95% CI) ^d^ | *P* ^d^ |
| --- | --- | --- | --- | --- | --- | --- | --- | --- | --- |
| *IL10* | rs3024496 | 1 | 205008487 | 3’URT | T/C | 3/95/916 | 3/99/893 | 0.96 (0.71-1.29) | 0.77 |
|  | rs1800871 | 1 | 205013257 | Intron | T/C | 128/426/440 | 127/402/442 | 1.05 (0.88-1.26) | 0.59 |
|  | rs1800896 | 1 | 205013520 | Intron | A/G | 13/162/837 | 10/166/813 | 0.98 (0.78-1.24) | 0.86 |
| *IL18R1* | rs3771167 | 2 | 102352620 | Intron | T/C | 2/67/948 | 0/72/926 | 0.92 (0.65-1.30) | 0.62 |
|  | rs1974675 | 2 | 102352807 | Intron | C/T | 22/214/781 | 18/252/728 | 0.81 (0.66-0.99) | **0.040** |
|  | rs6758936 | 2 | 102357801 | Intron | G/A | 19/246/745 | 19/256/712 | 0.92 (0.76-1.13) | 0.43 |
|  | rs6750020 | 2 | 102361146 | Intron | G/A | 211/468/328 | 210/503/273 | 0.78 (0.64-0.94) | **0.010** |
|  | rs1035130 | 2 | 102367834 | Exon 6 F251F | G/A | 94/417/499 | 100/438/459 | 0.85 (0.71-1.02) | 0.077 |
|  | rs3771158 | 2 | 102376326 | Intron | T/C | 10/160/846 | 12/191/794 | 0.78 (0.62-0.98) | **0.035** |
| *STAT1* | rs2280235 | 2 | 191552075 | Intron | C/T | 205/515/281 | 199/506/291 | 1.09 (0.90-1.33) | 0.37 |
|  | rs16833155 | 2 | 191569622 | Intron | C/T | 4/99/907 | 2/85/902 | 1.21 (0.89-1.64) | 0.22 |
|  | rs13029247 | 2 | 191574903 | Intron | C/T | 219/487/292 | 200/501/286 | 1.00 (0.82-1.22) | 0.99 |
|  | rs7576984 | 2 | 191576828 | Intron | C/A | 16/290/701 | 29/229/728 | 1.25 (1.03-1.52) | **0.027** |
|  | rs2066802 | 2 | 191582912 | Exon 1 L21L | T/C | 26/368/609 | 41/316/629 | 1.15 (0.96-1.38) | 0.14 |
| *IL12B* | rs1368439 | 5 | 158674592 | 3’URT | T/G | 0/5/1007 | 0/8/985 | 0.62 (0.20-1.92) | 0.40 |
|  | rs919766 | 5 | 158680142 | Intron | A/C | 4/98/910 | 2/94/894 | 1.01 (0.75-1.36) | 0.96 |
|  | rs3212217 | 5 | 158687708 | 3’URT | G/C | 188/494/329 | 191/464/342 | 1.08 (0.90-1.31) | 0.40 |
|  | rs2546892 | 5 | 158688053 | Intron | G/A | 44/314/604 | 46/289/653 | 1.17 (0.97-1.41) | 0.11 |
| *LTA* | rs2009658 | 6 | 31646223 | Intron | C/G | 29/290/685 | 28/241/720 | 1.25 (1.03-1.52) | **0.024** |
|  | rs1800683 | 6 | 31648050 | 5’UTR | G/A | 176/475/341 | 185/474/318 | 0.91 (0.75-1.10) | 0.32 |
|  | rs2229094 | 6 | 31648535 | Exon 1 C13R | T/C | 54/356/598 | 45/320/626 | 1.19 (0.99-1.43) | 0.061 |
|  | rs2229092 | 6 | 31648736 | Exon 2 H51P | A/C | 1/47/965 | 0/37/960 | 1.35 (0.87-2.11) | 0.18 |
|  | rs1041981 | 6 | 31648763 | Exon 2 T60N | C/A | 172/486/344 | 186/482/322 | 0.91 (0.76-1.10) | 0.34 |
| *TNF* | rs1800629 | 6 | 31651010 | Intron | G/A | 5/129/878 | 3/116/877 | 1.11 (0.85-1.45) | 0.45 |
|  | rs3093662 | 6 | 31652168 | Intron | A/G | 2/92/910 | 0/84/902 | 1.09 (0.80-1.49) | 0.57 |
| *IFNGR1* | rs1887415 | 6 | 137560931 | Exon 7 L467P | T/C | 0/63/953 | 2/60/935 | 0.98 (0.68-1.42) | 0.92 |
|  | rs2234711 | 6 | 137582213 | 5’UTR | C/T | 211/473/323 | 191/478/307 | 0.96 (0.79-1.16) | 0.66 |
| *IL27* | rs181206 | 16 | 28420904 | Exon 4 L119R | T/C | 11/232/757 | 20/235/734 | 0.93 (0.76-1.15) | 0.52 |
| *CCL2* | rs4586 | 16 | 29607382 | Exon 2 C35C | C/T | 157/452/395 | 161/477/354 | 0.86 (0.71-1.03) | 0.095 |
| *IL12RB1* | rs2305740 | 19 | 18041236 | Intron | A/G | 7/209/800 | 16/205/777 | 0.96 (0.77-1.18) | 0.68 |
|  | rs401502 | 19 | 18041413 | Exon 11 C378R | C/G | 117/463/428 | 109/460/416 | 1.00 (0.83-1.19) | 0.97 |
|  | rs375947 | 19 | 18041451 | Exon 11 M365T | A/G | 116/466/432 | 112/465/421 | 0.99 (0.83-1.18) | 0.91 |
|  | rs17852635 | 19 | 18047575 | Exon 7 P228P | G/A | 106/459/438 | 111/465/422 | 0.95 (0.80-1.14) | 0.58 |
|  | rs11575934 | 19 | 18047618 | Exon 7 Q214R | A/G | 112/457/429 | 108/458/418 | 0.98 (0.82-1.18) | 0.85 |
| *IFNGR2* | rs1059293 | 21 | 33731563 | 3’URT | T/C | 15/196/796 | 10/218/763 | 0.87 (0.71-1.08) | 0.21 |

**Table S2.** continued.

| Gene | SNP_ID | Allele ^b^ | OR (95% CI) ^e^ | *P* ^e^ | OR (95% CI) ^f^ | *P* ^f^ |
| --- | --- | --- | --- | --- | --- | --- |
| *IL10* | rs3024496 | T/C | 0.97 (0.19-4.86) | .97 | 0.96 (0.72-1.27) | 0.78 |
|  | rs1800871 | T/C | 0.99 (0.76-1.30) | .97 | 1.02 (0.90-1.17) | 0.71 |
|  | rs1800896 | A/G | 1.30 (0.56-3.00) | .54 | 1.00 (0.81-1.23) | 0.99 |
| *IL18R1* | rs3771167 | T/C | - | - | 0.94 (0.67-1.32) | 0.74 |
|  | rs1974675 | C/T | 1.17 (0.62-2.21) | .63 | 0.85 (0.71-1.03) | 0.090 |
|  | rs6758936 | G/A | 0.94 (0.49-1.80) | .86 | 0.93 (0.78-1.12) | 0.44 |
|  | rs6750020 | G/A | 0.95 (0.76-1.18) | .65 | 0.88 (0.78-1.00) | 0.054 |
|  | rs1035130 | G/A | 0.88 (0.66-1.19) | .42 | 0.89 (0.78-1.02) | 0.087 |
|  | rs3771158 | T/C | 0.80 (0.34-1.88) | .61 | 0.80 (0.65-0.99) | **0.039** |
| *STAT1* | rs2280235 | C/T | 1.04 (0.84-1.30) | .70 | 1.05 (0.93-1.20) | 0.42 |
|  | rs16833155 | C/T | 2.22 (0.40-12.46) | .35 | 1.22 (0.91-1.63) | 0.18 |
|  | rs13029247 | C/T | 1.10 (0.88-1.36) | .40 | 1.03 (0.91-1.17) | 0.62 |
|  | rs7576984 | C/A | 0.52 (0.28-0.97) | **.035** | 1.13 (0.95-1.34) | 0.18 |
|  | rs2066802 | T/C | 0.61 (0.37-1.01) | .051 | 1.06 (0.90-1.24) | 0.51 |
| *IL12B* | rs1368439 | T/G | - | - | - | - |
|  | rs919766 | A/C | 1.67 (0.30-9.22) | .55 | 1.02 (0.77-1.36) | 0.88 |
|  | rs3212217 | G/C | 0.96 (0.76-1.20) | .70 | 1.02 (0.90-1.16) | 0.73 |
|  | rs2546892 | G/A | 1.01 (0.66-1.54) | .98 | 1.11 (0.95-1.30) | 0.18 |
| *LTA* | rs2009658 | C/G | 1.04 (0.61-1.77) | .88 | 1.19 (1.00-1.41) | **0.045** |
|  | rs1800683 | G/A | 0.90 (0.71-1.13) | .35 | 0.93 (0.82-1.05) | 0.24 |
|  | rs2229094 | T/C | 1.18 (0.78-1.78) | .43 | 1.15 (0.99-1.34) | 0.065 |
|  | rs2229092 | A/C | - | - | 1.37 (0.89-2.12) | 0.15 |
|  | rs1041981 | C/A | 0.87 (0.69-1.09) | .23 | 0.92 (0.81-1.04) | 0.19 |
| *TNF* | rs1800629 | G/A | 1.49 (0.35-6.32) | .59 | 1.11 (0.86-1.43) | 0.41 |
|  | rs3093662 | A/G | - | - | 1.12 (0.82-1.52) | 0.48 |
| *IFNGR1* | rs1887415 | T/C | - | - | 0.94 (0.66-1.35) | 0.75 |
|  | rs2234711 | C/T | 1.10 (0.88-1.37) | .40 | 1.01 (0.89-1.15) | 0.85 |
| *IL27* | rs181206 | T/C | 0.57 (0.27-1.20) | .13 | 0.91 (0.76-1.10) | 0.33 |
| *CCL2* | rs4586 | C/T | 0.96 (0.75-1.22) | .72 | 0.92 (0.81-1.04) | 0.18 |
| *IL12RB1* | rs2305740 | A/G | 0.37 (0.15-0.91) | **.024** | 0.91 (0.75-1.11) | 0.36 |
|  | rs401502 | C/G | 1.06 (0.80-1.40) | .70 | 1.01 (0.88-1.16) | 0.87 |
|  | rs375947 | A/G | 1.02 (0.77-1.35) | .89 | 1.00 (0.87-1.14) | 0.98 |
|  | rs17852635 | G/A | 0.95 (0.71-1.26) | .70 | 0.96 (0.84-1.10) | 0.55 |
|  | rs11575934 | A/G | 1.03 (0.78-1.37) | .84 | 1.00 (0.87-1.14) | 0.97 |
| *IFNGR2* | rs1059293 | T/C | 1.47 (0.65-3.31) | .35 | 0.91 (0.75-1.11) | 0.36 |

Chr, chromosome; OR, odds ratio; CI, confidence interval. ^a^ Genomic position (NCBI Build 36). ^b^ Major allele / minor allele. ^c^ Number of minor homozygotes/number of heterozygotes/number of major homozygotes. *P* values, ORs and 95% CIs were calculated under dominant ^d^, recessive ^e^, and additive ^f^ genetic models by logistic regression while adjusting for age and sex. *P* value, OR and 95% CI were not available in some situations due to low frequency of SNPs.
