# Supplementary material for: Polymorphisms in the Interleukin 18 Receptor 1 Gene and Tuberculosis Susceptibility among Chinese
Source: PLoS One. 2014 Oct 31;9(10):e110734. doi: 10.1371/journal.pone.0110734 (PMC4216003; doi:10.1371/journal.pone.0110734)
Supplement: Table S3 — Chr, chromosome; OR, odds ratio; CI, confidence interval. a Major allele/minor allele. b Number of minor homozygotes/number of heterozygotes/number of major homozygotes. c P values, ORs and 95% CIs were calculated under dominant genetic models by logistic regression while adjusting for age and sex. d P heterogeneity were calculated to compare the difference of ORs within each stratum of age (<46 and ≥46 years). (DOCX) [file pone.0110734.s003.docx]

**Table S3.** Stratification analysis for 35 tag SNPs by age in the tuberculosis cases-control population.

| Gene | SNP_ID | Allele ^a^ | Age (years) | Cases ^b^ | Controls ^b^ | OR (95% CI) ^c^ | *P* ^c^ | *P* ^d^ |
| --- | --- | --- | --- | --- | --- | --- | --- | --- |
| IL10 | rs3024496 | T/C | ≥ 46 | 0/35/333 | 2/63/492 | 0.80 (0.51-1.23) | 0.30 | 0.18 |
|  |  |  | < 46 | 3/60/583 | 1/36/401 | 1.20 (0.78-1.86) | 0.40 |  |
|  | rs1800871 | T/C | ≥ 46 | 41/156/163 | 79/223/238 | 0.93 (0.71-1.22) | 0.60 | 0.30 |
|  |  |  | < 46 | 87/270/277 | 48/179/204 | 1.16 (0.90-1.49) | 0.24 |  |
|  | rs1800896 | A/G | ≥ 46 | 3/60/304 | 7/101/445 | 0.86 (0.61-1.22) | 0.41 | 0.23 |
|  |  |  | < 46 | 10/102/533 | 3/65/368 | 1.17 (0.84-1.65) | 0.35 |  |
| IL18R1 | rs3771167 | T/C | ≥ 46 | 0/18/351 | 0/38/520 | 0.76 (0.42-1.36) | 0.35 | 0.31 |
|  |  |  | < 46 | 2/49/597 | 0/34/406 | 1.04 (0.65-1.65) | 0.88 |  |
|  | **rs1974675** | **C/T** | **≥ 46** | **5/62/302** | **11/148/399** | **0.57 (0.41-0.79)** | **0.00050** | **0.0034** |
|  |  |  | **< 46** | **17/152/479** | **7/104/329** | **1.05 (0.79-1.39)** | **0.74** |  |
|  | **rs6758936** | **G/A** | **≥ 46** | **4/78/284** | **12/157/382** | **0.65 (0.47-0.88)** | **0.0053** | **0.0020** |
|  |  |  | **< 46** | **15/168/461** | **7/99/330** | **1.24 (0.93-1.64)** | **0.14** |  |
|  | rs6750020 | G/A | ≥ 46 | 65/168/133 | 109/282/160 | 0.73 (0.55-0.97) | 0.032 | 0.47 |
|  |  |  | < 46 | 146/300/195 | 101/221/113 | 0.84 (0.63-1.11) | 0.21 |  |
|  | rs1035130 | G/A | ≥ 46 | 28/155/184 | 45/239/274 | 0.97 (0.75-1.27) | 0.85 | 0.24 |
|  |  |  | < 46 | 66/262/315 | 55/199/185 | 0.79 (0.62-1.02) | 0.07 |  |
|  | rs3771158 | T/C | ≥ 46 | 2/48/319 | 8/114/435 | 0.69 (0.48-0.93) | 0.01 | 0.061 |
|  |  |  | < 46 | 8/112/527 | 4/77/359 | 0.99 (0.72-1.37) | 0.97 |  |
| STAT1 | rs2280235 | C/T | ≥ 46 | 91/192/81 | 105/293/158 | 1.46 (1.07-2.00) | 0.016 | 0.056 |
|  |  |  | < 46 | 114/323/200 | 94/213/133 | 0.95 (0.72-1.24) | 0.70 |  |
|  | rs16833155 | C/T | ≥ 46 | 2/42/321 | 1/47/504 | 1.49 (0.96-2.31) | 0.078 | 0.27 |
|  |  |  | < 46 | 2/57/586 | 1/38/398 | 1.00 (0.65-1.54) | 0.99 |  |
|  | rs13029247 | C/T | ≥ 46 | 79/192/91 | 109/285/156 | 1.16 (0.86-1.58) | 0.33 | 0.24 |
|  |  |  | < 46 | 140/295/201 | 91/216/130 | 0.93 (0.71-1.22) | 0.59 |  |
|  | rs7576984 | C/A | ≥ 46 | 6/110/249 | 19/139/393 | 1.11 (0.83-1.49) | 0.47 | 0.28 |
|  |  |  | < 46 | 10/180/452 | 10/90/335 | 1.44 (1.08-1.92) | 0.011 |  |
|  | rs2066802 | T/C | ≥ 46 | 10/139/216 | 25/179/345 | 1.16 (0.88-1.52) | 0.30 | 0.92 |
|  |  |  | < 46 | 16/229/393 | 16/137/284 | 1.19 (0.92-1.55) | 0.18 |  |
| IL12B | rs1368439 | T/G | ≥ 46 | 0/3/364 | 0/3/551 | 1.58 (0.31-8.13) | 0.58 | 0.10 |
|  |  |  | < 46 | /2/643 | /5/434 | 0.22 (0.04-1.17) | 0.059 |  |
|  | rs919766 | A/C | ≥ 46 | 1/31/335 | 1/49/503 | 0.93 (0.58-1.49) | 0.76 | 0.87 |
|  |  |  | < 46 | 3/67/575 | 1/45/391 | 1.03 (0.69-1.53) | 0.90 |  |
|  | rs3212217 | G/C | ≥ 46 | 72/172/124 | 104/260/194 | 1.07 (0.81-1.42) | 0.62 | 0.71 |
|  |  |  | < 46 | 116/322/205 | 87/204/148 | 1.11 (0.85-1.45) | 0.44 |  |
|  | rs2546892 | G/A | ≥ 46 | 18/120/208 | 24/161/368 | 1.32 (0.99-1.75) | 0.056 | 0.22 |
|  |  |  | < 46 | 26/194/396 | 22/128/285 | 1.03 (0.79-1.34) | 0.84 |  |
| LTA | rs2009658 | C/G | ≥ 46 | 14/106/246 | 19/136/398 | 1.23 (0.92-1.64) | 0.17 | 0.94 |
|  |  |  | < 46 | 15/184/439 | 9/105/322 | 1.27 (0.97-1.68) | 0.085 |  |
|  | rs1800683 | G/A | ≥ 46 | 65/166/126 | 82/271/190 | 0.98 (0.74-1.31) | 0.91 | 0.37 |
|  |  |  | < 46 | 111/309/215 | 103/203/128 | 0.80 (0.61-1.05) | 0.10 |  |
|  | rs2229094 | T/C | ≥ 46 | 21/131/214 | 27/186/341 | 1.14 (0.87-1.51) | 0.33 | 0.55 |
|  |  |  | < 46 | 33/225/384 | 18/134/285 | 1.28 (0.99-1.65) | 0.061 |  |
|  | rs2229092 | A/C | ≥ 46 | 1/22/344 | 0/23/535 | 1.52 (0.83-2.77) | 0.18 | 0.67 |
|  |  |  | < 46 | /25/621 | /14/425 | 1.21 (0.61-2.40) | 0.57 |  |
|  | rs1041981 | C/A | ≥ 46 | 62/173/128 | 83/275/195 | 1.00 (0.76-1.33) | 0.97 | 0.29 |
|  |  |  | < 46 | 110/313/216 | 103/207/127 | 0.77 (0.59-1.01) | 0.062 |  |
| TNF | rs1800629 | G/A | ≥ 46 | 3/46/319 | 0/61/496 | 1.21 (0.80-1.82) | 0.36 | 0.44 |
|  |  |  | < 46 | 2/83/559 | 3/55/381 | 1.00 (0.70-1.45) | 0.99 |  |
|  | rs3093662 | A/G | ≥ 46 | 1/26/337 | 0/45/504 | 0.99 (0.59-1.63) | 0.96 | 0.35 |
|  |  |  | < 46 | 1/66/573 | 0/39/398 | 1.25 (0.82-1.91) | 0.30 |  |
| IFNGR1 | rs1887415 | T/C | ≥ 46 | 0/26/343 | 0/31/527 | 1.25 (0.72-2.16) | 0.43 | 0.26 |
|  |  |  | < 46 | 0/37/610 | 2/29/408 | 0.83 (0.50-1.38) | 0.47 |  |
|  | rs2234711 | C/T | ≥ 46 | 77/159/129 | 112/260/174 | 0.89 (0.67-1.19) | 0.44 | 0.29 |
|  |  |  | < 46 | 134/314/194 | 79/218/133 | 1.10 (0.84-1.45) | 0.47 |  |
| IL27 | rs181206 | T/C | ≥ 46 | 4/78/279 | 12/138/403 | 0.82 (0.60-1.12) | 0.20 | 0.14 |
|  |  |  | < 46 | 7/154/478 | 8/97/331 | 1.06 (0.80-1.42) | 0.67 |  |
| CCL2 | rs4586 | C/T | ≥ 46 | 63/161/140 | 93/267/195 | 0.87 (0.66-1.15) | 0.32 | 0.83 |
|  |  |  | < 46 | 94/291/255 | 68/210/159 | 0.85 (0.65-1.09) | 0.20 |  |
| IL12RB1 | rs2305740 | A/G | ≥ 46 | 2/66/301 | 4/122/432 | 0.82 (0.58-1.14) | 0.23 | 0.12 |
|  |  |  | < 46 | 5/143/499 | 12/83/345 | 1.07 (0.80-1.45) | 0.64 |  |
|  | rs401502 | C/G | ≥ 46 | 44/160/161 | 57/274/219 | 0.85 (0.65-1.11) | 0.24 | 0.084 |
|  |  |  | < 46 | 73/303/267 | 52/186/197 | 1.17 (0.91-1.50) | 0.22 |  |
|  | rs375947 | A/G | ≥ 46 | 42/164/161 | 59/275/224 | 0.87 (0.67-1.14) | 0.32 | 0.15 |
|  |  |  | < 46 | 74/302/271 | 53/190/197 | 1.13 (0.88-1.45) | 0.34 |  |
|  | rs17852635 | G/A | ≥ 46 | 38/158/164 | 59/275/224 | 0.81 (0.62-1.07) | 0.14 | 0.093 |
|  |  |  | < 46 | 68/301/274 | 52/190/198 | 1.10 (0.86-1.42) | 0.45 |  |
|  | rs11575934 | A/G | ≥ 46 | 42/158/163 | 57/271/220 | 0.83 (0.63-1.09) | 0.19 | 0.077 |
|  |  |  | < 46 | 70/299/266 | 51/187/198 | 1.16 (0.90-1.49) | 0.25 |  |
| IFNGR2 | rs1059293 | T/C | ≥ 46 | 7/71/287 | 4/121/429 | 0.91 (0.65-1.26) | 0.55 | 0.55 |
|  |  |  | < 46 | 8/125/509 | 6/97/334 | 0.86 (0.63-1.15) | 0.31 |  |

Chr, chromosome; OR, odds ratio; CI, confidence interval. ^a^ Major allele/minor allele. ^b^ Number of minor homozygotes/number of heterozygotes/number of major homozygotes. ^c^ *P* values, ORs and 95% CIs were calculated under dominant genetic models by logistic regression while adjusting for age and sex. ^d^ *P*_heterogeneity_ were calculated to compare the difference of ORs within each stratum of age (< 46 and ≥ 46 years).
