# Supplementary material for: Polymorphisms in the Interleukin 18 Receptor 1 Gene and Tuberculosis Susceptibility among Chinese
Source: PLoS One. 2014 Oct 31;9(10):e110734. doi: 10.1371/journal.pone.0110734 (PMC4216003; doi:10.1371/journal.pone.0110734)
Supplement: Table S4 — a Differences of mean age between the protective allele carriers and the at-risk homozygotes were analyzed by use of an unpaired t test. The protective alleles are T and A for rs1974675 and rs6758936 respectively. (DOCX) [file pone.0110734.s004.docx]

Table S4: The association between genotypes of rs1974675 and rs6758936 and age at TB diagnosis in the older patients (≥ 46 years).

| Polymorphism | n (%) | Age, mean in years ±SD | *P*^a^ |
| --- | --- | --- | --- |
| rs1974675 |  |  |  |
| C/C | 302 (81.8) | 61.3 ± 11.0 |  |
| C/T | 62 (16.8) | 62.8 ± 13.4 |  |
| T/T | 5 (1.4) | 57.8 ± 8.6 |  |
| C/T-T/T | 67 (18.2) | 62.4 ± 13.1 | 0.52 |
| rs6758936 |  |  |  |
| G/G | 284 (77.6) | 61.6 ± 11.2 |  |
| G/A | 78 (21.3) | 60.7 ± 12.1 |  |
| A/A | 4 (1.1) | 67.3 ± 6.7 |  |
| G/A-A/A | 82 (22.4) | 61.1 ± 12.0 | 0.72 |

^a^ Differences of mean age between the protective allele carriers and the at-risk homozygotes were analyzed by use of an unpaired *t* test. The protective alleles are T and A for rs1974675 and rs6758936 respectively.
