# Supplementary material for: Polymorphisms in the Interleukin 18 Receptor 1 Gene and Tuberculosis Susceptibility among Chinese
Source: PLoS One. 2014 Oct 31;9(10):e110734. doi: 10.1371/journal.pone.0110734 (PMC4216003; doi:10.1371/journal.pone.0110734)
Supplement: Table S5 — When loaded with an index SNP, SNPinfo can include SNPs with strong linkage disequilibrium (LD) with it and predicts those that may affect following biological functions with alternative allele: transcriptional regulation by affecting transcription factor binding sites (TFBS) activity; premature termination of amino-acid sequence (stop codons); changing of splicing pattern or efficiency by disrupting splice site, exonic splicing enhancers (ESE) or silencers (ESS); alteration of protein structures or properties by changing single amino acids or changing the frame of the protein-coding region; regulation of protein translation by affecting microRNA (miRNA) binding sites activity, and etc (http://snpinfo.niehs.nih.gov/snpinfo). None of the five coding SNPs in strong LD (r2>0.8, CHB data) with rs1974675 in this locus were predicted functional. None of the fifteen non-coding SNPs were predicted to alter transcriptional factor binding site, splicing, or miRNA binding site. (DOCX) [file pone.0110734.s005.docx]

**Table S5.** The predicted function of rs1974675 and 20 SNPs in strong linkage disequilibrium with it using SNPinfo programme.

| No. | SNPs | Position | Allele | Nearby Gene | Distance (bp) | TFBS | Splicing | | |  | miRNA | | nsSNP | Stop Codon | Polyphen | SNPs3D | | RegPotential | Conservation |
| --- | --- | --- | --- | --- | --- | --- | --- | --- | --- | --- | --- | --- | --- | --- | --- | --- | --- | --- | --- |
|  |  |  |  |  |  |  | site | ESE or ESS | abolish domain |  | miRanda | Sanger |  |  |  | svm profile | svm structure |  |  |
| 1 | rs10208293 | 102332742 | A/G | IL1RL1 | 38348\|\|2187 | - | - | - | - |  | - | - | - | - | - | - | - | 0 | 0 |
| 2 | rs1861245 | 102333338 | T/C | IL1RL1 | 38944\|\|1591 | - | - | - | - |  | - | - | - | - | - | - | - | 0 | 0 |
| 3 | rs13424006 | 102333668 | C/T | IL1RL1 | 39274\|\|1261 | - | - | - | - |  | - | - | - | - | - | - | - | 0 | 0 |
| 4 | rs6751967 | 102333845 | C/T | IL1RL1 | 39451\|\|1084 | - | - | - | - |  | - | - | - | - | - | - | - | 0 | 0 |
| 5 | rs6749114 | 102334019 | A/C | IL1RL1 | 39625\|\|910 | - | - | - | - |  | - | - | - | - | - | - | - | NA | 0.001 |
| 6 | rs4988955 | 102334360 | A/G | IL1RL1 | 39966\|\|569 | - | - | - | - |  | - | - | - | - | - | - | - | 0 | 0.001 |
| 7 | rs4988956 | 102334439 | A/G | IL1RL1 | 40045\|\|490 | - | - | Y | Y |  | - | - | Y | - | benign | - | - | 0.016 | 0.162 |
| 8 | rs4988957 | 102334507 | C/T | IL1RL1 | 40113\|\|422 | - | - | - | - |  | - | - | - | - | - | - | - | 0.48 | 0.017 |
| 9 | rs10192036 | 102334643 | A/C | IL1RL1 | 40249\|\|286 | - | - | - | - |  | - | - | Y | - | benign | - | - | 0.29 | 0.028 |
| 10 | rs10204137 | 102334644 | A/G | IL1RL1 | 40250\|\|285 | - | - | - | - |  | - | - | Y | - | benign | - | - | 0.30 | 0.029 |
| 11 | rs4988958 | 102334717 | C/T | IL1RL1 | 40323\|\|212 | - | - | - | - |  | - | - | - | - | - | - | - | 0.16 | 0.985 |
| 12 | rs10192157 | 102334788 | C/T | IL1RL1 | 40394\|\|141 | - | - | - | - |  | - | - | Y | - | benign | - | - | 0.082 | 0 |
| 13 | rs10206753 | 102334794 | C/T | IL1RL1 | 40400\|\|135 | - | - | - | - |  | - | - | Y | - | benign | - | - | 0.12 | 0.002 |
| 14 | rs7603730 | 102340803 | A/C | IL1RL1\|\|IL18R1 | -5874\|\|-4726 | - | - | - | - |  | - | - | - | - | - | - | - | 0 | 0 |
| 15 | rs10170583 | 102341196 | A/G | IL1RL1\|\|IL18R1 | -6267\|\|-4333 | - | - | - | - |  | - | - | - | - | - | - | - | 0 | 0 |
| 16 | rs10176664 | 102342604 | A/G | IL1RL1\|\|IL18R1 | -7675\|\|-2925 | - | - | - | - |  | - | - | - | - | - | - | - | 0 | 0.002 |
| 17 | rs10204837 | 102344162 | A/C | IL1RL1\|\|IL18R1 | -9233\|\|-1367 | - | - | - | - |  | - | - | - | - | - | - | - | 0.028 | 0 |
| 18 | rs3755276 | 102344891 | T/C | IL1RL1\|\|IL18R1 | -9962\|\|-638 | - | - | - | - |  | - | - | - | - | - | - | - | 0.046 | 0 |
| 19 | rs1362348 | 102351056 | G/C | IL18R1 | 5527\|\|30594 | - | - | - | - |  | - | - | - | - | - | - | - | 0 | 0 |
| 20 | rs3771166 | 102352654 | G/A | IL18R1 | 7125\|\|28996 | - | - | - | - |  | - | - | - | - | - | - | - | 0 | 0 |
| 21 | rs1974675 | 102352807 | G/A | IL18R1 | 7278\|\|28843 | - | - | - | - |  | - | - | - | - | - | - | - | 0 | 0.001 |

When loaded with an index SNP, SNPinfo can include SNPs with strong linkage disequilibrium (LD) with it and predicts those that may affect following biological functions with alternative allele: transcriptional regulation by affecting transcription factor binding sites (TFBS) activity; premature termination of amino-acid sequence (stop codons); changing of splicing pattern or efficiency by disrupting splice site, exonic splicing enhancers (ESE) or silencers (ESS); alteration of protein structures or properties by changing single amino acids or changing the frame of the protein-coding region; regulation of protein translation by affecting microRNA (miRNA) binding sites activity, and etc (<http://snpinfo.niehs.nih.gov/snpinfo>). None of the five coding SNPs in strong LD (*r^2^* > 0.8, CHB data) with rs1974675 in this locus were predicted functional. None of the fifteen non-coding SNPs were predicted to alter transcriptional factor binding site, splicing, or miRNA binding site.
